# Notes on two *Stiphropus* species from China (Araneae, Thomisidae)

**DOI:** 10.3897/BDJ.11.e105695

**Published:** 2023-06-02

**Authors:** Fengyuan Li, Yejie Lin, Shuqiang Li

**Affiliations:** 1 State Key Laboratory of Protein and Plant Gene Research, School of Life Sciences, Peking University, Beijing 100871, China State Key Laboratory of Protein and Plant Gene Research, School of Life Sciences, Peking University Beijing 100871 China; 2 Hebei Key Laboratory of Animal Diversity, College of Life Science, Langfang Normal University, Langfang 065000, China Hebei Key Laboratory of Animal Diversity, College of Life Science, Langfang Normal University Langfang 065000 China; 3 Institute of Zoology, Chinese Academy of sciences, Beijing 100101, China Institute of Zoology, Chinese Academy of sciences Beijing 100101 China

**Keywords:** diagnosis, new species, misidentification, taxonomic, myrmecophilous

## Abstract

**Background:**

The spider genus *Stiphropus* Gerstaecker, 1873 currently includes 21 extant species that are distributed in Africa (12) and Asia (9). Four species, *S.falciformus* Yang, Zhu & Song, 2006, *S.myrmecophilus* Huang & Lin, 2020, *S.ocellatus* Thorell, 1887 and *S.soureni* Sen, 1964, are currently known from China.

**New information:**

The mismatched female of *S.falciformus* is reported as a new species: *S.qianlei* sp. n. (♂♀). The unknown male of *S.soureni* Sen, 1964 is described for the first time. Photos and morphological descriptions are provided.

## Introduction

The crab spider family Thomisidae Sundevall, 1833 contains 171 genera and 2172 known species worldwide (World Spider Catalog 2023). The subfamily Stiphropodinae Simon, 1895 includes three genera: *Stiphropus* Gerstaecker, 1873, *Stiphropella* Lawrence, 1952 and *Heterogriffus* Platnick, 1976, which possess peg-like setae on the chelicerae (Benjamin 2011), *Stiphropus* with the type species *S.lugubris* Gerstaecker, 1873, described from east Africa, being one of its easily recognised taxa. The type species can be distinguished by the stout and fused metatarsus and tarsus with densely plumose hairs (Ono 1980).

*Stiphropus* currently comprises 21 species, distributed in Asia and Africa. Recently, a large number of new spider species have been reported from China (Li 2020, Li et al. 2021, Yao et al. 2021, Lu et al. 2022, Zhao et al. 2022), but *Stiphropus* is poorly studied in the region. Until now, only four species are known from China, three species are endemic, whereas *S.ocellatus* Thorell, 1887 is recorded from China, Myanmar and Vietnam (Sen 1964, Ono 1980, Yang et al. 2006, Zhu and Shan 2007, Huang and Lin 2020).

During the examination of *Stiphropus* specimens from China (Guizhou, Tibet and Yunnan), we found the undescribed male of *S.soureni* Sen, 1964 from Tibet and determined that the supposed female of *S.falciformus* Yang, Zhu & Song, 2006 described from Guizhou is mismatched and, in actuality, constitutes a new species: *S.qianlei* sp. n. is the new species and is closely allied with the ant species *Aphaenogastersmythiesii* (Forel, 1902). The goal of this paper is to provide description of this new species and the undescribed female of *S.soureni*.

## Materials and methods

All specimens were preserved in 80% ethanol. The spermathecae were cleared in trypsin enzyme solution to dissolve non-chitinous tissues. Specimens were examined under a Leica M205C stereomicroscope. Photomicrographs were taken with an Olympus C7070 zoom digital camera (7.1 megapixels). Laboratory habitus photographs were taken with a Sony A7RIV digital camera, equipped with a Sony FE 90mm Goss lens. Photos were stacked with Helicon Focus® (Version 7.6.1) or Zerene Stacker® (Version 1.04) and processed in Adobe Photoshop CC2022®. The distribution map was generated with ArcGIS v. 10.2 (ESRI Inc.).

All measurements are in millimetres (mm) and were obtained with an Olympus SZX16 stereomicroscope with a Zongyuan CCD industrial camera. All measurements of body lengths do not include the chelicerae. Eye sizes are measured as the maximum diameter from either the dorsal or frontal view. Leg measurements are given as follows: total length (femur, patella, tibia, metatarsus, tarsus). The type materials are deposited in the Institute of Zoology, Chinese Academy of Sciences in Beijing (**IZCAS**).

Abbreviations: **AG** accessory gland; **ALE** anterior lateral eye; **AME** anterior median eye; **CD** copulatory duct; **CO** copulatory opening; **DTA** dorsal tibial apophysis; **E** embolus; **FD** fertilisation duct; **PLE** posterior lateral eye; **PME** posterior median eye; **S** spermatheca; **Tu** tutaculum; **VTA** ventral tibial apophysis.

## Taxon treatments

### Stiphropus qianlei

Lin & Li, 2023 sp. n.

53C6E257-9DE3-586A-B36C-A64DE3F64437

3076D183-D5D6-40E4-A150-10CC5960F0B1

#### Materials

- **Type status:** Holotype. **Occurrence:** recordedBy: Hao Yu, Qianle Lu; individualCount: 1; sex: male; lifeStage: adult; occurrenceID: A91C5F74-DF5B-507E-87FD-266101905E4F; **Taxon:** scientificName: *Stiphropusqianlei*; **Location:** country: China; stateProvince: Guizhou; county: Xiuwen; locality: Liutong Town, Weijiayakou; verbatimElevation: 1109 m; verbatimCoordinates: 27.09°N, 106.50°E; **Identification:** identifiedBy: Yejie Lin; dateIdentified: 2023; **Event:** year: 2022; month: 6; day: 5; habitat: ant nest; **Record Level:** institutionCode: IZCAS-Ar44580
- **Type status:** Paratype. **Occurrence:** recordedBy: Hao Yu, Qianle Lu; individualCount: 1; sex: female; lifeStage: adult; occurrenceID: A4F5873C-8D16-5E40-A9DE-635752986206; **Taxon:** scientificName: *Stiphropusqianlei*; **Location:** country: China; stateProvince: Guizhou; county: Xiuwen; locality: Liutong Town, Weijiayakou; verbatimElevation: 1109 m; verbatimCoordinates: 27.09°N, 106.50°E; **Identification:** identifiedBy: Yejie Lin; dateIdentified: 2023; **Event:** year: 2022; month: 6; day: 5; habitat: ant nest; **Record Level:** institutionCode: IZCAS-Ar44581
- **Type status:** Paratype. **Occurrence:** recordedBy: Hao Yu, Qianle Lu; individualCount: 1; sex: female; lifeStage: adult; occurrenceID: 48F30C8A-7113-5C52-9CDF-1E915D45A80F; **Taxon:** scientificName: *Stiphropusqianlei*; **Location:** country: China; stateProvince: Guizhou; county: Xiuwen; locality: Liutong Town, Weijiayakou; verbatimElevation: 1109 m; verbatimCoordinates: 27.09°N, 106.50°E; **Identification:** identifiedBy: Yejie Lin; dateIdentified: 2023; **Event:** year: 2022; month: 7; day: 4; habitat: ant nest; **Record Level:** institutionCode: IZCAS-Ar44582
- **Type status:** Paratype. **Occurrence:** recordedBy: Hao Yu, Qianle Lu; individualCount: 1; sex: female; lifeStage: adult; occurrenceID: 96767AD9-BDD2-57D4-AF88-2F745D5D219C; **Taxon:** scientificName: *Stiphropusqianlei*; **Location:** country: China; stateProvince: Guizhou; county: Xiuwen; locality: Liutong Town, Weijiayakou; verbatimElevation: 1109 m; verbatimCoordinates: 27.09°N, 106.50°E; **Identification:** identifiedBy: Yejie Lin; dateIdentified: 2023; **Event:** year: 2022; month: 7; day: 4; habitat: ant nest; **Record Level:** institutionCode: IZCAS-Ar44583

#### Description

Male (holotype). Total length 2.71; carapace 1.29 long, 1.12 wide, opisthosoma 1.55 long, 1.16 wide. Eye sizes and interdistances: AME 0.06, ALE 0.11, PME 0.02, PLE 0.09, AME–AME 0.07, AME–ALE 0.17, PME–PME 0.17, PME–PLE 0.26, AME–PME 0.07, ALE–PLE 0.13. Chelicerae with seven promarginal spines. Endites 3 times longer than wide. Leg measurements: I 3.13 (0.86, 0.56, 0.92, 0.79), II 3.12 (0.84, 0.92, 0.54, 0.82), III 2.29 (0.68, 0.79, 0.34, 0.48), IV 2.38 (0.71, 0.81, 0.37, 0.49).

Colouration (Fig. 5a). Carapace dark-brown, covered with long sparse brown setae, glaborous posteriorly. Endites and labium brown. Sternum yellowish-brown. Legs brown to dark-brown, covered with plumose setae, more obvious in metatarsi and tarsi. Opisthosoma brown, covered with scutum, with long, sparse brown setae. Spinnerets yellow.

**Figure 1.**
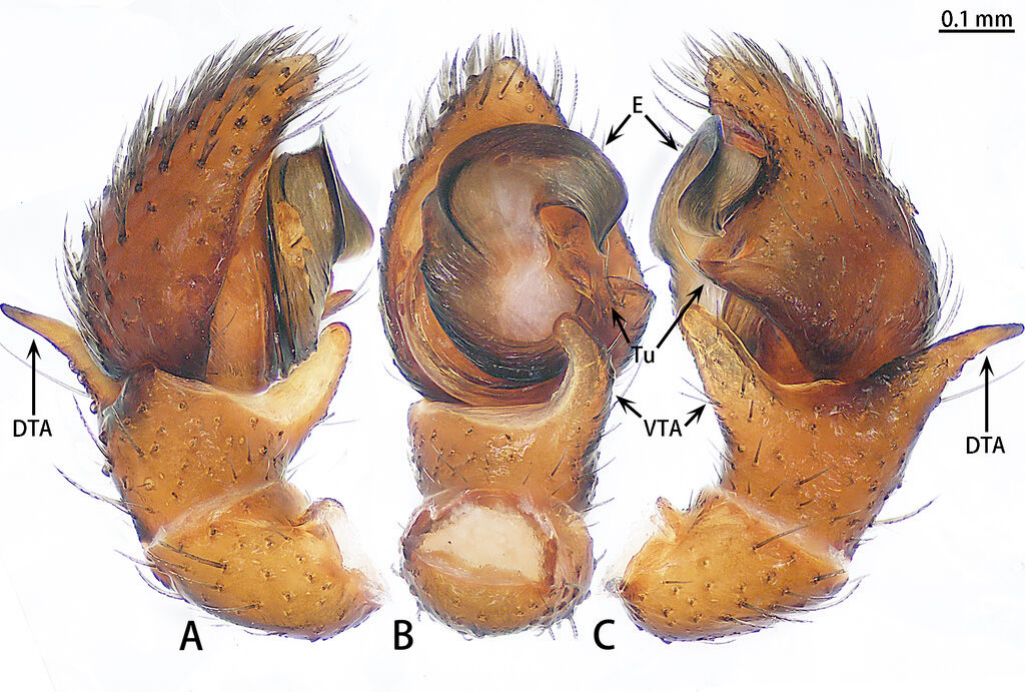
*Stiphropusqianlei* sp. n., holotype male. **A** Prolateral view; **B** Ventral view; **C** Retrolateral view. Abbreviations: **DTA** dorsal tibial apophysis; **E** embolus; **Tu** tutaculum; **VTA** ventral tibial apophysis.

Palp (Fig. 1). Patella almost as long as tibia. Tibia with 2 apophyses, ventral tibial apophysis blunt, little curved; dorsal tibial apophysis as long as ventral tibial apophysis, terminal sharp. Cymbium almost as long as wide. Cymbium covered with sparse plumose setae at dorso-prolateral half. Tutaculum with two sub-triangular projections in ventral view. Tegulum crescent-shaped. Embolus falciform, wrinkled and rounded distally in retrolateral view, widest at the middle, originating from 11:30 o’clock position and its tip ending at 3:00 o’clock position, opening dorsally at tip.

Female (IZCAS-Ar44581). Total length 4.57; carapace 1.80 long, 1.45 wide, opisthosoma 2.64 long, 2.49 wide. Eye sizes and interdistances: AME 0.07, ALE 0.13, PME 0.02, PLE 0.13, AME–AME 0.09, AME–ALE 0.21, PME–PME 0.23, PME–PLE 0.34, AME–PME 0.13, ALE–PLE 0.16. Chelicerae with seven promarginal spines. Endites 3 times longer than wide. Leg measurements: I 3.73 (0.99, 1.07, 0.60, 1.07), II 3.85 (1.05, 1.14, 0.60, 1.06), III 3.06 (0.92, 1.06, 0.42, 0.66), IV 3.33 (1.02, 1.11, 0.53, 0.67).

Colouration (Fig. 5a). Similar to that of male, except paler and dorsal with two large muscle dots.

**Figure 2.**
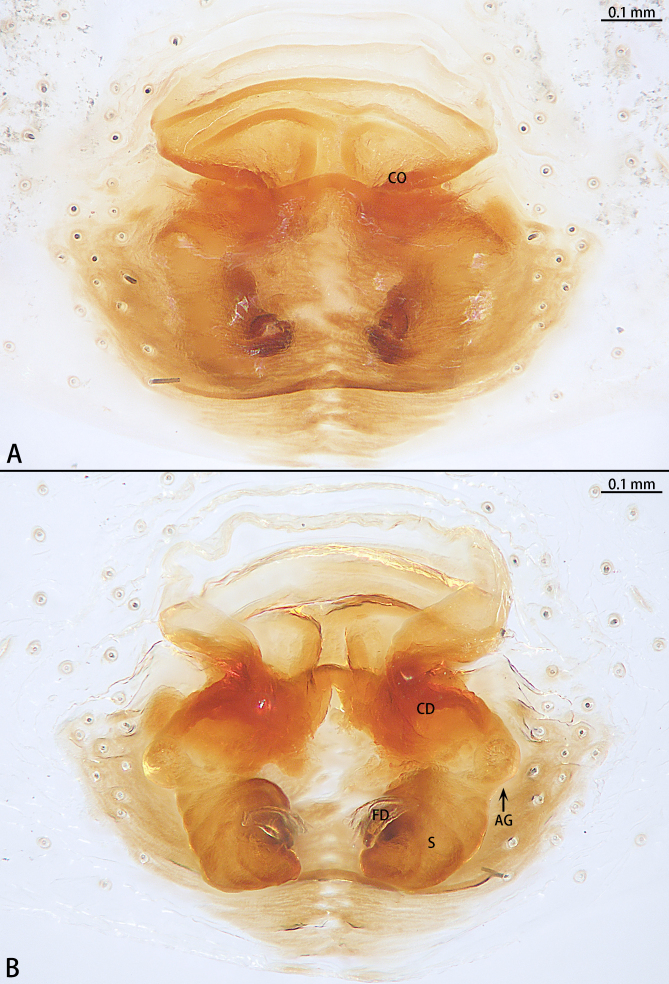
*Stiphropusqianlei* sp. n., paratype female. **A** Epigyne, ventral view; **B** Vulva, dorsal view. Abbreviations: **AG** accessory gland; **CD** copulatory duct; **CO** copulatory opening; **FD** fertilisation duct; **S** spermatheca; **Tu** tutaculum.

Epigyne (Fig. 2) raised near middle of the abdomen, with a transversal, sub-oval and wrinkled depression anteriorly, two creviced and arched copulatory openings located at sides of posterior margin of the wrinkled depression, touching each other. Copulatory ducts strongly sclerotised at parts next to ducts and almost as long as spermathecae. Spermathecae almost spherical, with irregularly distributed projections on the surface, spaced by less than 1 radius. Fertilisation ducts originated near mesal sides of spermathecae.

#### Diagnosis

The male is similar to *S.ocellatus* Thorell, 1887 by the curved and wide embolus and long, terminal sharp dorsal tibial apophysis (see Ono 1980, figs. 22–27). However, the new species can be distinguished from *S.ocellatus* by the embolus strongly curved as C-shaped and widest at the middle [vs. no significant change in the width of the embolus in *S.ocellatus* (Ono 1980, figs. 22 and 25) and dorsal tibial apophysis pointed dorsally [vs. point anteriorly in *S.ocellatus* (Ono 1980, figs, 23, 24, 26 and 27). For diagnosis of females, see Li et al. (2010).

#### Etymology

The species is named after one of the collectors.

#### Distribution

China (Guizhou) (Fig. 7).

#### Biology

All specimens were collected in two different nests of *Aphaenogastersmythiesii* (Forel, 1902). *S.qianlei* sp. n. can be classified as a myrmecophile or myrmecophage. The collectors did not search for *S.qianlei* sp. n. outside the ant nests, but evidence suggests that the discovery of *S.qianlei* sp. n. in ant nests is typical (Fig. 6).

#### Notes

The oval fan-shaped folds, the 90° folded copulatory duct and accessory gland point posteriorly show the females of *S.falciformus* in Li et al. (2010) are mismatched. Herein, we treated their female taxon as *S.qianlei* sp. n.

#### Compared material examined

*Stiphropusfalciformus* 1♂, CHINA: *Yunnan*: Xishuangbanna, Mengla County, Menglun Town, Menglun Nature Reserve, Guo Zheng leg.

### Stiphropus soureni

Sen, 1964

F5DFFCB8-39ED-5026-9F0A-474197EB03D5

52RSZ

#### Materials

- **Type status:** Holotype. **Occurrence:** recordedBy: Shri S. Biswas; individualCount: 1; sex: female; lifeStage: adult; occurrenceID: 80E3E6C2-C7B3-50D7-A130-DAD83EBB5051; **Taxon:** scientificName: *Stiphropussoureni*; **Location:** country: China; stateProvince: Tibet; county: Lhoka; locality: Cona City, Dalongzong, Morshing (Mursing) Village; verbatimCoordinates: 27.1644°N, 92.2161°E; **Identification:** identifiedBy: J. K. Sen; dateIdentified: 1964; identificationRemarks: not examined; **Event:** year: 1961; month: 9; day: 7; **Record Level:** institutionID: Zoological Survey India; institutionCode: Calcutta 2805/18
- **Type status:** Other material. **Occurrence:** recordedBy: Zhuo Chen; individualCount: 1; sex: male; lifeStage: adult; occurrenceID: BF77FA44-4378-5C82-A84E-78595BCA4025; **Taxon:** scientificName: *Stiphropussoureni*; **Location:** country: China; stateProvince: Tibet; county: Nyingchi; locality: Medog County, Jiangxin Village; verbatimCoordinates: 29.2001°N, 95.1246°E; **Identification:** identifiedBy: Yejie Lin; dateIdentified: 2017; **Event:** year: 2015; month: 8; day: 15; habitat: under bark; **Record Level:** institutionCode: IZCAS-Ar44584
- **Type status:** Other material. **Occurrence:** recordedBy: Zhuo Chen; individualCount: 1; sex: female; lifeStage: adult; occurrenceID: BDBF7D3E-8D76-5E3F-A306-9739D018B9EA; **Taxon:** scientificName: *Stiphropussoureni*; **Location:** country: China; stateProvince: Tibet; county: Nyingchi; locality: Medog County, Jiangxin Village; verbatimCoordinates: 29.2001°N, 95.1246°E; **Identification:** identifiedBy: Yejie Lin; dateIdentified: 2017; **Event:** year: 2015; month: 8; day: 15; habitat: under bark; **Record Level:** institutionCode: IZCAS-Ar44585

#### Description

Male. Total length 3.43; carapace 1.59 long, 1.44 wide, opisthosoma 1.80 long, 1.85 wide. Eye sizes and interdistances: AME 0.05, ALE 0.07, PME 0.02, PLE 0.06, AME–AME 0.05, AME–ALE 0.11, PME–PME 0.13, PME–PLE 0.18, AME–PME 0.06, ALE–PLE 0.08. Chelicerae with seven promarginal spines. Endites 3 times longer than wide. Leg measurements: I 3.41 (0.84, 1.09, 0.58, 0.90), II 3.53 (0.92, 1.15, 0.59, 0.87), III 2.71 (0.79, 0.95, 0.44, 0.53), IV 2.71 (0.87, 0.87, 0.44, 0.53).

Colouration (Fig. 5b). Carapace dark-brown, covered with long sparse brown setae, glabrous posteriorly. Endites and labium dark-brown. Sternum brown. Legs dark-brown, covered with plumose setae, more obvious in metatarsi and tarsi. Opisthosoma orange, covered scutum, with long, sparse brown setae and seven obvious muscle dots. Spinnerets yellow.

**Figure 3.**
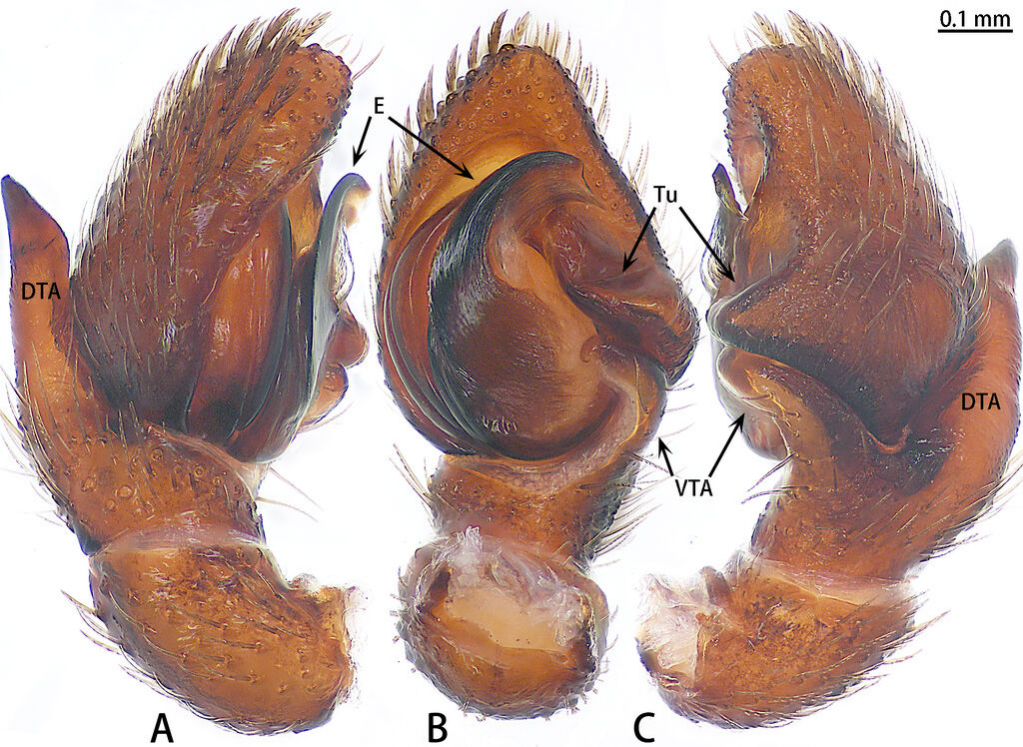
*Stiphropussoureni*, male. **A** Prolateral view; **B** Ventral view; **C** Retrolateral view. Abbreviations: **DTA** dorsal tibial apophysis; **E** embolus; **Tu** tutaculum; **VTA** ventral tibial apophysis.

Palp (Fig. 3). Patella almost as long as tibia. Tibia with two apophyses, ventral tibial apophysis blunt, little curved; dorsal tibial apophysis almost two times longer than ventral tibial apophysis, terminal sharp. Cymbium slightly longer than wide (1:1.2). Cymbium covered with sparse plumose setae at dorso-prolateral half, with a sub-triangular projection adjacent to the tutaculum in retrolateral view. Tegulum crescent-shaped. Embolus falciform, wrinkled, terminal sharp in ventral view, widest at the middle, originating from 11:30 o’clock position and its tip ending at 2:00 o'clock position.

**Figure 4.**
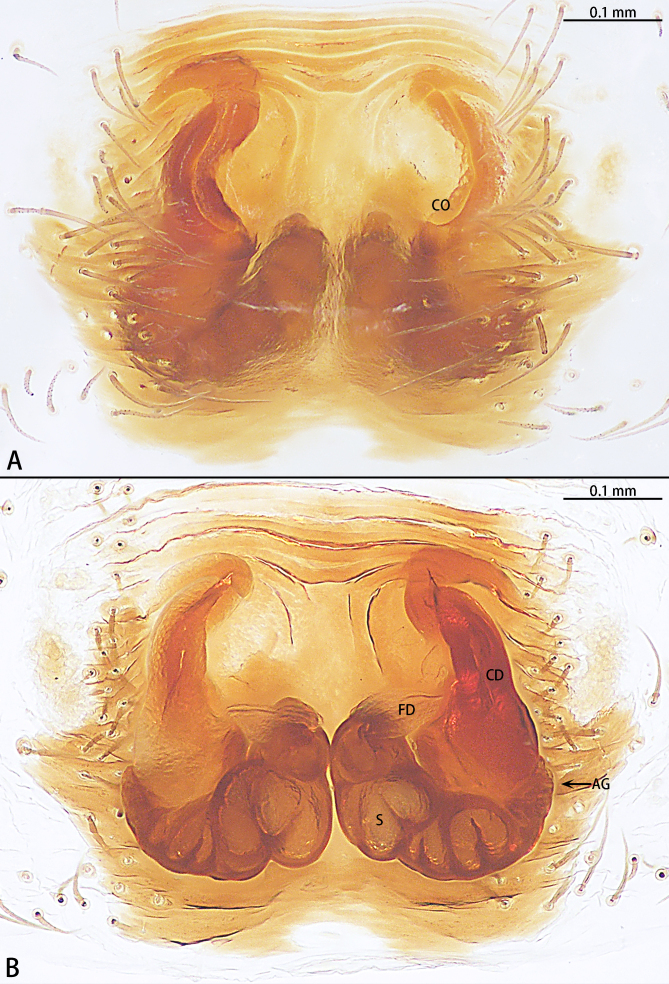
*Stiphropussoureni* female. **A** Epigyne, ventral view; **B** Vulva, dorsal view. Abbreviations: **AG** accessory gland; **CD** copulatory duct; **CO** copulatory opening; **FD** fertilisation duct; **S** spermatheca; **Tu** tutaculum.

Female. See Ono (1980) (Fig. 4 and Fig. 5b). Large scuta around the pair of largest muscle dots.

#### Diagnosis

The male is similar to that of *S.ocellatus* Thorell, 1887 by the curved embolus, terminal sharp and long, strong dorsal tibial apophysis (See Ono (1980), figs. 22–27). However, the new species can be distinguished from *S.ocellatus* by the embolus pointed to 1:30 o’clock position and its tip ending at 2:00 o'clock position [vs. pointed to 2:00 o’clock position and its tip ending at 3:00 o’clock position in *S.ocellatus* (Ono (1980), figs. 22 and 25) and dorsal tibial apophysis pointed prolaterally [vs. point anteriorly in *S.ocellatus* (Ono (1980), figs. 23, 24, 26 and 27). For the diagnosis of females, see Ono (1980). In habitus, *S.soureni* sp. n. with orange opisthosoma in male (Fig. 5b) (black in *S.ocellatus*).

#### Distribution

**Figure 7.**
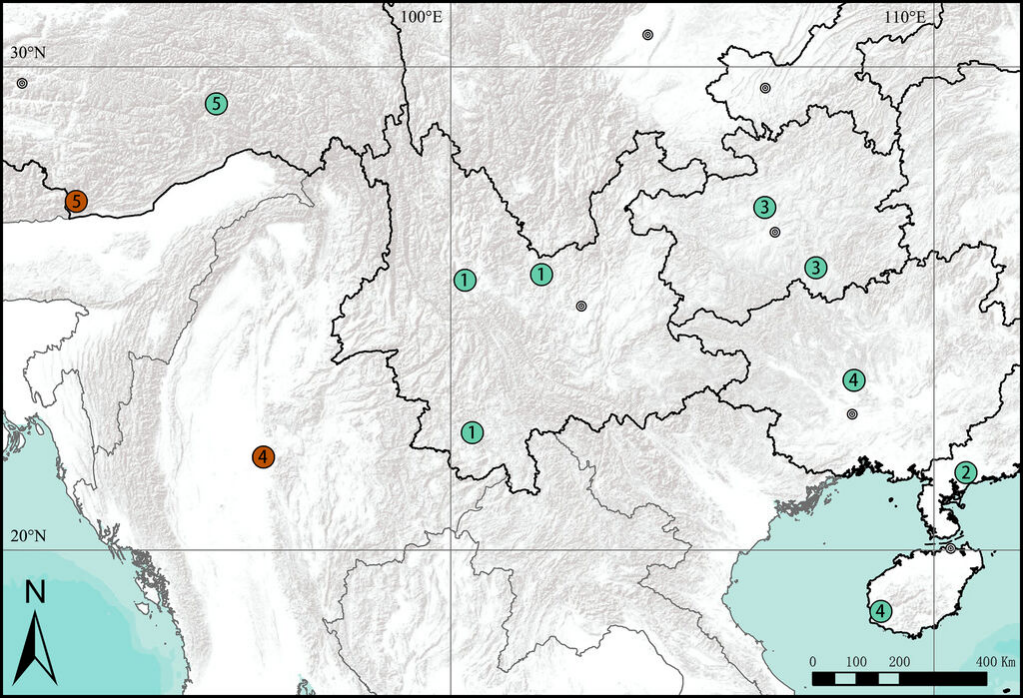
Distribution records of *Stiphropus* species in Asia: **1** *Stiphropusfalciformus*; **2** *S.myrmecophilus*; **3** *S.qianlei* sp. n.; **4** *S.ocellatus*; **5** *S.soureni*. Brown circles shows the holotypes collection located.

China (Tibet) (Fig. 7).

#### Biology

This species live under the bark of trees.

#### Notes

The male is described here for the first time.

## Supplementary Material

XML Treatment for Stiphropus qianlei

XML Treatment for Stiphropus soureni

## Figures and Tables

**Figure 5a.**
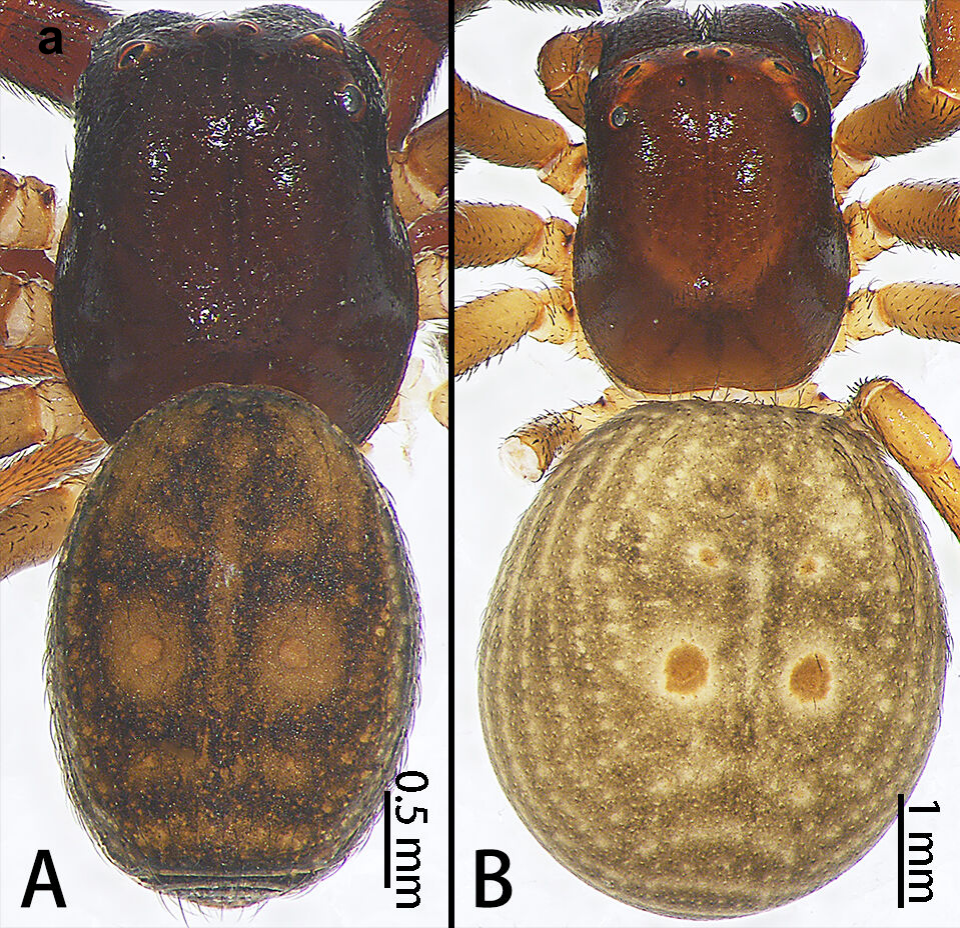
*Stiphropusqianlei* sp. n.;

**Figure 5b.**
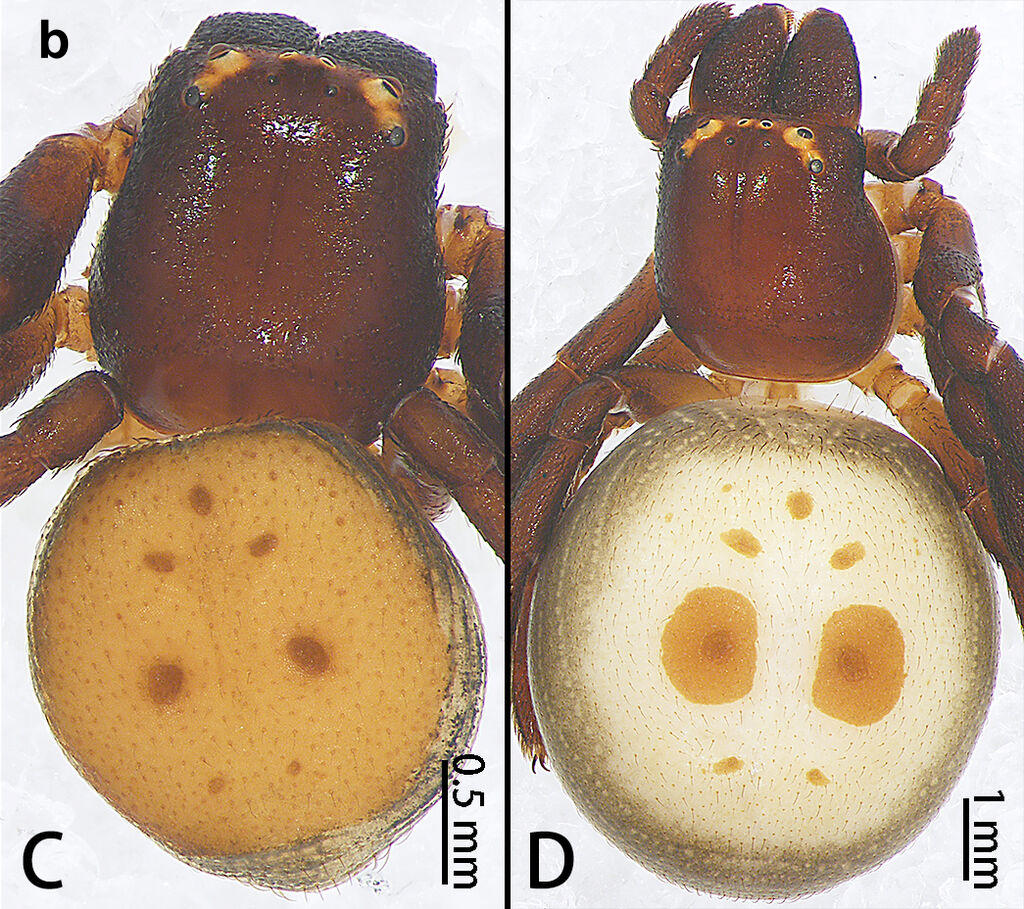
*S.soureni*.

**Figure 6a.**
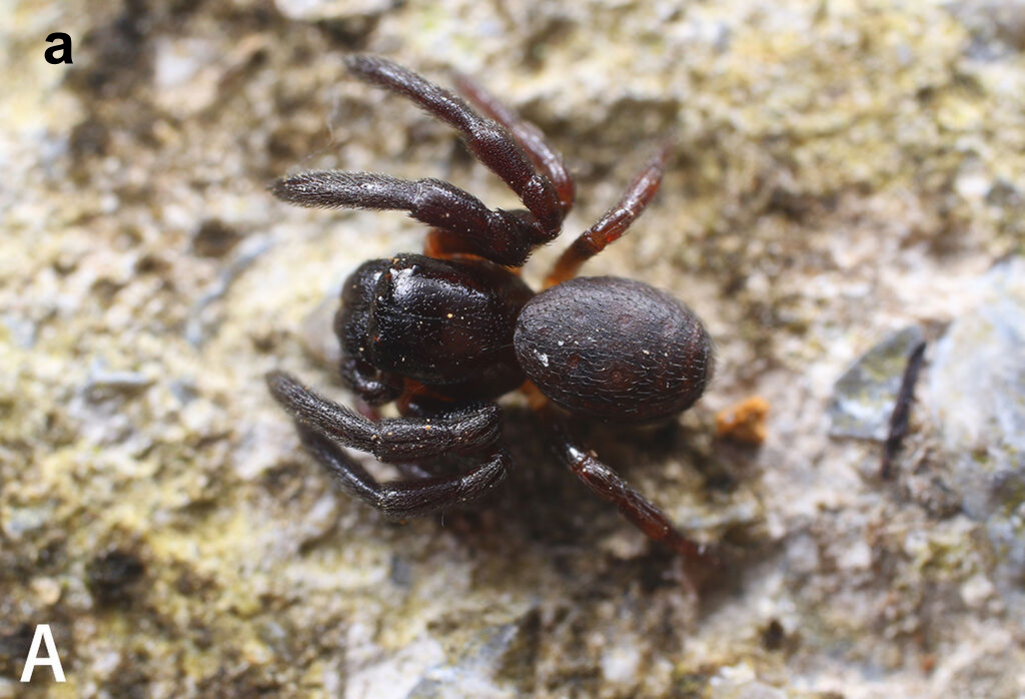
Holotype male;

**Figure 6b.**
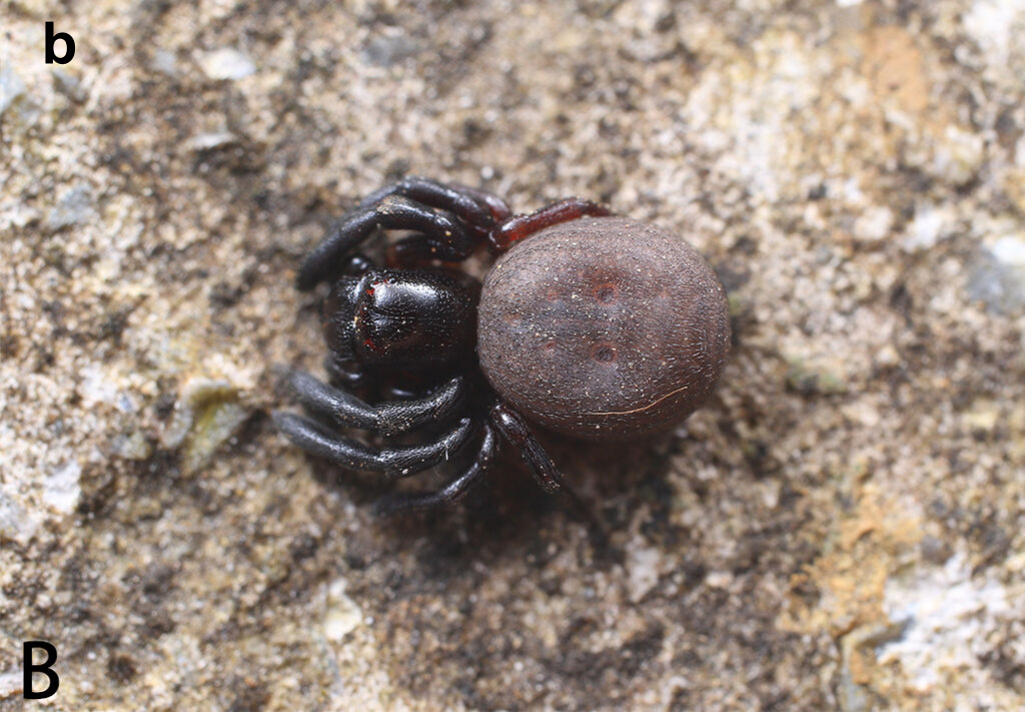
Paratype female;

**Figure 6c.**
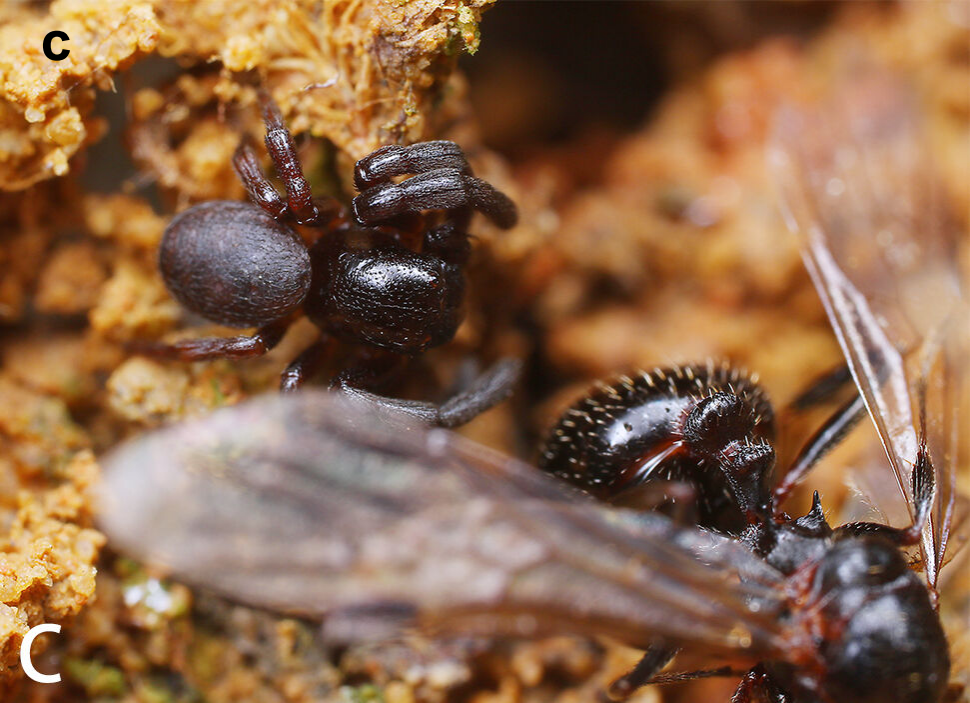
Holotype male with ant;

**Figure 6d.**
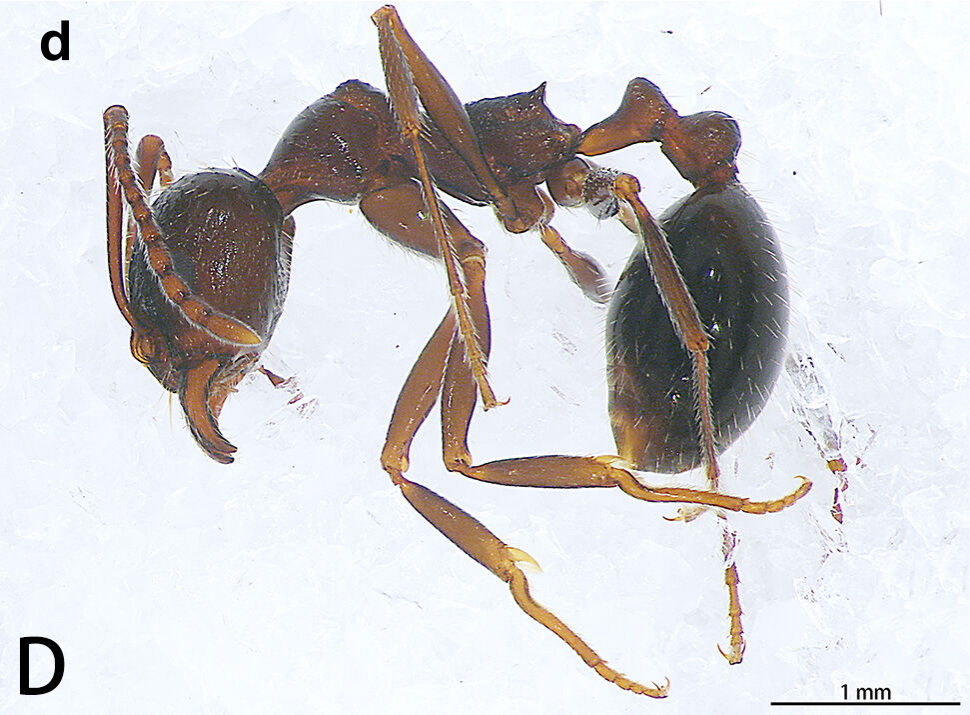
Host ant *Aphaenogastersmythiesii*.
